# Clinical Applications of Circulating Tumor Cells in Lung Cancer Patients by CellSearch System

**DOI:** 10.3389/fonc.2014.00242

**Published:** 2014-09-04

**Authors:** Anna Truini, Angela Alama, Maria Giovanna Dal Bello, Simona Coco, Irene Vanni, Erika Rijavec, Carlo Genova, Giulia Barletta, Federica Biello, Francesco Grossi

**Affiliations:** ^1^Lung Cancer Unit, IRCCS AOU San Martino-IST, Genoa, Italy

**Keywords:** circulating tumor cells, lung cancer, biomarker, prognostic marker, predictive marker

## Abstract

Circulating tumor cells (CTCs) are cells spread from the primary tumor into the bloodstream that might represent an important biomarker in lung cancer. The prognosis of patients diagnosed with lung cancer is generally poor mainly due to late diagnosis. Recent evidences have reported that tumor aggressiveness is associated with the presence of CTCs in the blood stream; therefore, several studies have focused their attention on CTC isolation, characterization, and clinical significance. So far, the CellSearch^®^ system is the only approach approved by FDA for metastatic breast, prostate, and colorectal cancer intended to detect CTCs of epithelial origin in whole blood and to assess prognosis. To date, no specific biomarkers have been validated in lung cancer and the identification of novel tumor markers such as CTCs might highly contribute to lung cancer prognosis and management. In the present review, the significance of CTC detection in lung cancer is examined through the analysis of the published studies in both non-small cell and small cell lung cancers; additionally the prognostic and the clinical role of CTC enumeration in treatment monitoring will be reported and discussed.

## CTC and Lung Cancer

Circulating tumor cells (CTCs) are widely recognized to be shed into the peripheral blood from solid tumor playing an important role in the development of metastasis (1, 2). Recent improvements in technical approaches able to identify CTCs from whole blood withdrawal have demonstrated the potential value of CTC detection as a liquid biopsy especially in those tumors where tissue accessibility is often challenging as in lung cancer (3, 4). Lung cancer is the first cause of cancer related death worldwide and both histotypes: small cell lung cancer (SCLC) and non-small cell lung cancer (NSCLC) have poor survival rate.

Although SCLC is more responsive to chemotherapy and radiotherapy than other types of lung cancers, it is frequently disseminated by the time of diagnosis and complete tumor response is generally difficult to achieve (median survival ranges from 7 to 12 months) (5). Similarly, the overall survival (OS) of NSCLC patients is generally poor (median survival of 12–14 months) and despite significant advances in the NSCLC management the 5-years OS remains unsatisfied (~15%) primarily attributable to late diagnosis when surgery is no longer possible (6).

The current standard of care for both advanced SCLC and NSCLC patients consists in platinum-based combination chemotherapy (7, 8) but the identification of activating mutations in the epidermal growth factor receptor (*EGFR*) gene and the presence of an anaplastic lymphoma kinase (*ALK*) rearrangement in sub-groups of NSCLC have improved the clinical outcome of these patients by treatment with specific tyrosine kinase inhibitors (i.e., gefitinib, erlotinib, afatinib) and *ALK* inhibitors (crizotinib, ceritinib), compared with conventional chemotherapy alone (9–12). Additionally, the possibility of performing analyses by means of CTCs, such as genotyping and molecular characterization, has opened new important perspectives for lung cancer patients by tailoring therapeutic strategies and monitoring treatment efficacy (13).

To date, the CellSearch^®^ (Veridex LLC, Raritan, NJ, USA) system is the only technique, approved by the US Food and Drug Administration (FDA), for baseline CTC enumeration as an aid to prognosis and treatment efficacy in breast, colorectal and prostate cancers (14–16). Although the CellSearch procedure needs to be further validated in lung cancer, a number of studies already reported significant data about the role of CTCs in this disease.

**Figure 1:**
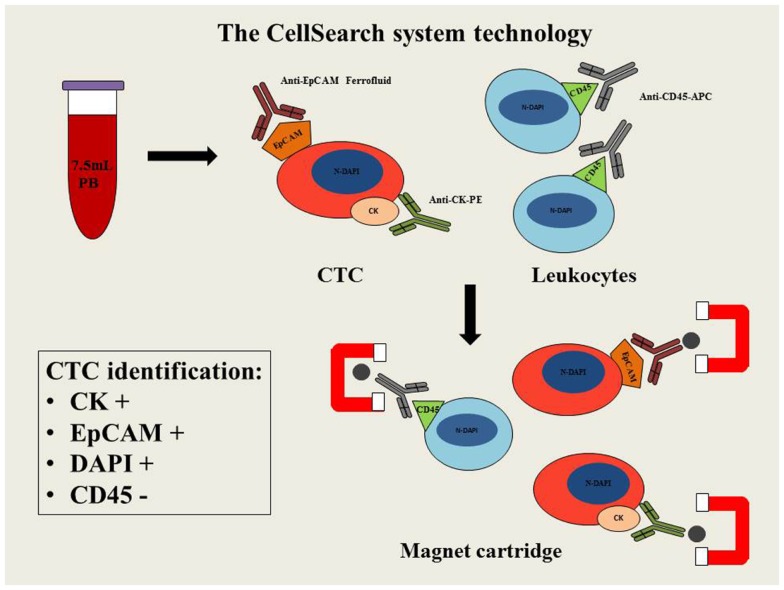
**Schematic diagram of CellSearch system technology**. PB, peripheral blood; CTC, circulating tumor cell; EpCAM, epithelial cell adhesion molecule; CK–PE, cytokeratins–phytoerythrin; CD45-APC, cluster of differentiation 45-allophycocyanin; N-DAPI, nucleus stained with DAPI.

The CellSearch system is a semi-automated methodology able to detect and enumerate CTCs. Analysis is carried out in 7.5 ml of whole blood using three different antibodies: the epithelial cell adhesion molecule (EpCAM), cytokeratin (CK), and CD45. Peripheral blood is mixed with magnetic iron nanoparticles coated with anti-EpCAM antibody to confer magnetic properties to the epithelial cells. In order to detect these epithelial cells and exclude the presence of leukocytes (CD45+), anti-CK and anti-CD45 fluorescent antibodies are respectively used. In addition cell nuclei are fluorescently labeled with the DAPI nuclear dye (4′,6-diamidino-2-phenylindole) to allow microscopic identification of the relevant cell fraction. A strong magnetic field is then applied to separate the cell population that can be detected and counted by digital fluorescent microscopy. The identification of CTCs is based on specific features: CK+, nuclear staining by DAPI and lack of CD45 expression (Figure 1). However, this system is unable to detect the CTCs, with fibroblastic-like phenotype (EpCAM-negative), that have been observed during disease progression and metastasis.

While several methodologies have been described to isolate the CTCs in lung cancer, the current review will focus on the most significant data in SCLC and NSCLC patients obtained by the CellSearch system.

One of the first study dates back to 2007 and reported the changes in CTC count before and after surgery in nine male patients who underwent surgery for NSCLC. The results showed that one patient and three patients reported CTCs prior to surgery and immediately after the operation, respectively and no CTCs were detected in any of these patients 10 days after surgery. Additionally no recurrence was reported after a median follow-up of 14 months in any of the patients, in concordance with CTC count (17).

Starting from the above-mentioned pioneering study in this field several investigations have focused on CTCs to explore their role in more detail (Table 1).

**Table 1:** **Summary of relevant results from CTC studies in lung cancer by CellSearch**.

Reference	Sample number	Histotype	Results
Sawabata et al. (17)	9	NSCLC	CTC-positive in 4/9 patients. No recurrence after surgery
Bevilacqua et al. (19)	5	SCLC	CTC have a role in SCLC patients diagnosis
Tanaka et al. (20)	150	NSCLC and SCLC	CTC well differentiate stage I from stage IV tumors
Hou et al. (21)	50	SCLC	CTCs ≥ 2 in 86%. Higher CTC number → shorter OS. CTC-positive decreased after CT (60%)
Naito et al. (22)	51	SCLC	CTCs detected in 68.6, 26.5, and 67.6% at B, post-CT, and R, respectively. CTCs > 8 → worse OS
Hiltermann et al. (23)	59	SCLC	Median CTCs at B = 6 and 63 in localized and metastatic disease, respectively. CTCs decrease after CT → strong predictor of OS
Normanno et al. (24)	60	SCLC	CTCs identified in 90% at B and associated to involved organs. CTC reduction higher than 89% after CT → better OS
Hou et al. (25)	97	SCLC	CTCs present in 85%. CTCs ≥ 50 at B → worse OS. Failure of CTC to decrease <50 after CT → worse prognosis
Krebs et al. (26)	101	NSCLC	CTC number at B higher in stage IV than stage III (60 and 27, respectively).CTCs number → prognostic factor for PFS and predictor of OS after first cycle of CT
Hirose et al. (27)	33	NSCLC	CTCs positive in 36.4 and 15.2% had five or more CTCs before CT. Positive CTCs patients → progressive disease
Punoose et al. (28)	41	NSCLC	CTCs identified in 78% at B. CTC decrease during CT → longer PFS
Muinelo-Romay et al. (29)	43	NSCLC	CTCs detected in 41.9%. CTCs ≥ 5 → shorter PFS and OS. CTC decrease after second cycle of CT → higher response and better PFS and OS
Isobe et al. (30)	24	NSCLC	CTCs detected in 33.3%. *EGFR* mutations in cfDNA: 25%. EGFR mutation rate in cfDNA higher when CTCs ≥ 2
Swennenhuis et al. (31)	10	NSCLC and SCLC	DNA isolation and sequencing from a single CTC

*NSCLC, non-small cell lung cancer; SCLC, small cell lung cancer; OS, overall survival; PFS, progression-free survival; B, baseline; CT, chemotherapy; R, relapse; cfDNA, circulating free DNA*.

## Role of CTC in Diagnosis and Prognosis

To date, several studies have shown that, in the absence of tumor biopsies, the detection of CTCs in the peripheral blood of patients with lung cancer may hold great promise as a surrogate tissue in defining prognosis and predicting efficacy of chemotherapy treatment (3, 4, 18).

The majority of patients with a pulmonary nodule undergo fine needle biopsy and are diagnosed by cytological examination only. However, extensive immune-cytochemical analyses are rarely feasible due to the small amount of available material, leading to misdiagnosis. Bevilacqua and colleagues performed analyses on peripheral blood sample using the CellSearch system in a patient diagnosed with low-grade neuroendocrine tumor following fine needle biopsy. EpCAM and CK positive expressions could not correlate with the neuroendocrine tumor diagnosis since no evidence of epithelial markers was found in this malignancy. Further immunophenotypic characterization on the biological material derived from a biopsy of a liver metastasis disclosed diffuse cell positivity for EpCAM and CK pool: these observations were consistent with a diagnosis of SCLC (19). Although this study underlined the importance of CTC in diagnosis of critic samples, the majority of the reported articles focused their attention on the prognostic role of CTCs.

A prospective study evaluating CTCs in 150 patients with a suspicious or a diagnosis of primary lung cancer using the CellSearch system was reported by Tanaka *et al*. The authors investigated the diagnostic role of CTCs by discriminating lung cancer from non-malignant disease. However, although CTC count was higher in lung cancer patients compared to patients with non-malignant disease, the receiver operating characteristic (ROC) curve did not disclose a good discrimination between lung cancer patients and healthy controls (CTCs were identified in 30.6 and 12%, respectively). Nevertheless, this study reported the significant role of CTCs in predicting distant metastasis (AUC-ROC, 0.783; *p* < 0.001) since CTC count was higher in SCLC compared to NSCLC and was able to well differentiate stage I from stage IV tumors (20).

Another article reporting the prognostic role of CTC has been published by Hou and colleagues who focused on the expression and clinical significance of CTCs in 50 patients affected by SCLC who received standard chemotherapy treatment (21). Baseline CTCs ≥2 cells were found in 86% of patients with a median CTC number of 28. The median survival for patients with CTC number >300 was 134 days compared with about 400 days for patients with low CTC number (*p* < 0.005); higher CTC number was significantly associated with shorter survival in the univariate analysis (*p* = 0.01) underlining the role of CTC in prognosis. In addition, in the 24 samples available after chemotherapy, the CTC number decreased following treatment being the CTCs detectable in 60% of patients only compared with 86% at baseline. These results provided a rationale to include CTC analysis in SCLC patients.

More recently, a study by Naito *et al*. showed the prospective assessment of the optimal CTC-cut-off, determined by CellSearch, to predict the OS in 51 patients with SCLC treated with chemotherapy or chemo-radiotherapy. Two or more CTCs were identified in 68.6, 26.5, and 67.6% of the patients at baseline, post-chemotherapy, and at relapse, respectively. The optimal cut-off of >8 CTCs per 7.5 ml of blood was recognized as significantly predictive of the OS and the CTC presence at post-treatment and at relapse strongly correlated with worse outcome (22).

Interestingly, an important study able to demonstrate that CTCs in SCLC represent a better predictor of survival than disease stage and tumor response, assessed by computed tomography (CT), was reported by Hiltermann and colleagues in 2012. Blood samples were obtained from 59 SCLC patients and CTCs were measured before, after one cycle, and at the end of chemotherapy. Lower numbers of CTCs at baseline were detected in patients with localized SCLC (median = 6) respect to patients with metastatic disease (median = 63). Additionally, a better clinical outcome was correlated with lack of measurable CTCs (*p* ≤ 0.001). CTCs decreased after the first cycle of chemotherapy and the multivariate Cox regression analysis revealed that CTC count was a stronger predictor for OS (*p* = 0.004) than disease stage and CT evaluation. Although CTC enumeration was not associated with tumor response after four cycles of chemotherapy, patients with low baseline CTC numbers lived longer than those with higher CTCs (23).

Recently, a study by Normanno and colleagues assessed the prognostic value of CTCs in 60 patients with extensive SCLC. Using the CellSearch system, the CTCs were enumerated and analyzed separately at baseline, after one cycle of chemotherapy and as a change between the two counts. CTCs were identified in 90% of the patients and the count was significantly associated with the number of organs involved by disease. CTC determination after one cycle of chemotherapy was not always feasible and the analyses between the two counts were performed in 40 patients only. Interestingly, a CTC reduction higher than 89% after chemotherapy was significantly associated with a better outcome. The authors concluded that an early decline of CTC number after the first chemotherapy cycle was significantly more useful than baseline CTC count in estimating prognosis in this sub-group of patients with advanced SCLC (24).

The prognostic value of CTC enumeration was further demonstrated by Hou and colleagues in 97 patients affected by SCLC receiving chemotherapy. They investigated the role of CTCs and CTC cluster, named circulating tumor microemboli (CTM). CTCs were detected in 85% of the patients at baseline (prior to any chemotherapy) whereas CTM were detected in 32% of the patients presenting CTCs. Categorizing patients into favorable and unfavorable group, based on CTC number, the authors reported a significantly different outcome in term of progression-free survival (PFS: 4.6 and 8.8 months, respectively), and OS (5.4 and 11.5 months, respectively). These results demonstrated that baseline CTC presence was an independent prognostic factor (*p* < 0.0001). CTC counts was the most significant variable in a multivariate model and failure of CTC number to decrease to less than 50 after one cycle of chemotherapy was associated with worse prognosis. This study demonstrated the presence of CTM in SCLC and that CTCs and CTM are associated with worse prognosis in SCLC (25).

In 2011, Krebs and colleagues reported the prognostic role of CTCs also in NSCLC. In a single-center prospective study, 101 chemo-naïve stage III–IV NSCLC patients were enrolled and evaluated before and after one cycle of chemotherapy. Twenty-one per cent of the patients had a CTC count at baseline greater than two cells and CTC number was much higher in stage IV patients compared to stage III NSCLC patients, reporting a mean of 60 and 27 CTCs, respectively. The univariate analysis showed a significantly better PFS and OS (*p* < 0.001) in patients with fewer CTCs. Furthermore, in the multivariate analysis, the CTC number remained an independent prognostic factor for PFS and was the strongest predictor of OS also in the sub-group of patients whose CTC analysis was feasible before and after one cycle of chemotherapy (26).

## Clinical Significance of CTC to Treatment Response

Besides the significance of the CTCs as diagnostic and prognostic indicators, an important goal for clinical application is represented by the early indication of patient response to conventional treatment.

Hirose and colleagues prospectively evaluated the relationship between CTC count and chemotherapy treatment in 33 metastatic NSCLC patients further treated with gemcitabine and carboplatin. This study revealed no differences in chemotherapy response between CTC-positive and CTC-negative patients whereas PFS was significantly higher in CTC-positive compared to CTC-negative patients revealing that CTCs might be an important predictive factor for the effectiveness of chemotherapy treatment in advanced NSCLC patients (27).

A multicenter phase II study, involving 41 advanced NSCLC patients treated with erlotinib and pertuzumab, investigated the predictive significance of CTC number variation during treatment in relation to response (evaluated with positron emission tomography and CT-scan) and clinical outcome. CTCs were identified at baseline in 78% of the patients, and a statistically significant correlation between high CTC count and treatment response was reported (*p* = 0.009). Additionally, decreased CTC count during treatment correlated with longer PFS (*p* = 0.05). These data suggest that CTC enumeration during therapy might help defining clinical response and monitoring treatment efficacy (28).

In another study, the blood samples of 43 advanced NSCLC patients were analyzed before the first, second, and fifth cycles of platinum-based chemotherapy. Morphologically intact CTCs were detected in 41.9% of the patients and patients with CTCs ≥5 presented a significantly shorter median PFS and OS (4.1 and 4.6 months, respectively) compared to patients with <5 CTCs (median PFS and OS of 7.6 and 10.7 months, respectively). A marked decrease of CTCs after the second cycle of chemotherapy correlated with an overall response rate and a better OS and PFS supporting the predictive role of CTCs as indicator of chemotherapy response (29).

## Future Perspectives

Alongside enumeration, the importance of CTC detection also relies on the possibility of performing molecular investigations in cluster or single CTCs to be correlated with data from the primary tumor tissue or circulating free DNA (cfDNA).

Isobe and colleagues evaluated the blood content of CTCs to determine a correlation between CTCs and cfDNA in advanced NSCLC patients after acquisition of resistance to EGFR tyrosine kinase inhibitors. CTCs were detected in 33.3% of the patients whereas *EGFR* mutations were reported in 25% of cases using the Cycleave real-time PCR assay. The *EGFR* mutation rate in cfDNA was significantly higher in patients with more than two CTCs per 7.5 ml of blood compared to patients reporting less than two CTCs, leading to hypothesize that the presence of *EGFR* mutation in cfDNA correlated with a higher number of CTCs in the blood and a more aggressive disease (30).

Based on the increased importance of CTCs, very recently Swennenhuis and colleagues evaluated efficiency and quality of DNA isolation from a single CTC in order to characterize the mutational landscape of CTCs. Using the CellSearch system followed by fluorescence-activated cell sorting (FACS), a single CTC was sequenced using whole genome amplification (WGA) reporting for the first time that 55% of the exome was successfully sequenced at 20× depth calling 72% of the variants (31).

## Conclusion

To date, the CellSearch system is the only method approved by FDA to detect and enumerate the CTCs in breast, colorectal, and prostate cancers but a number of studies in SCLC and NSCLC have shown that CTCs might represent an important diagnostic and prognostic biomarker in lung cancer as well. Additionally, an important goal for clinical application involves the early indication of patient’s response to conventional treatment by analyzing CTC variation.

Blood samples can be obtained on a serial basis during treatment allowing the identification of early chemotherapy resistance profiles in real time. Indeed variations in CTC levels during treatment may reveal changes in disease status that might influence therapeutic decisions in the clinical setting.

Although the timing of CTC detection and imaging evaluations during treatment, reported in various studies, are rather heterogeneous, additional data about CTC enumeration associated with standard imaging techniques, in determining diagnosis and prognosis of lung cancer, need to be warranted.

The feasibility to carry out molecular characterization of CTCs using innovative sequencing technologies may additionally provide more specific and informative mutational landscape of the cells involved in distant metastasis and recognize early recurrence or chemotherapy resistance in lung cancer patients.

## Author Contributions

Francesco Grossi, Angela Alama and Anna Truini conceptualized the idea for this review. Francesco Grossi, Angela Alama, Anna Truini, Simona Coco, and Irene Vanni participated in the design of the review. Anna Truini, Angela Alama, Maria Giovanna Dal Bello, and Simona Coco collected and read the related papers for the manuscript. Erika Rijavec, Carlo Genova, Giulia Barletta, and Federica Biello performed data retrieval on the relationship between CTC and clinical significance. All authors substantially contributed to the manuscript, drafting the work and revising all the sections critically. All authors read and approved the final version of the manuscript.

## Conflict of Interest Statement

The authors declare that the research was conducted in the absence of any commercial or financial relationships that could be construed as a potential conflict of interest.
